# Hepatic Hilum Variations and Their Clinical Considerations in the Liver: A Systematic Review and Meta-Analysis

**DOI:** 10.3390/life14101301

**Published:** 2024-10-14

**Authors:** Juan Jose Valenzuela-Fuenzalida, Fernanda Pena-Santibañez, Ayline Vergara Salinas, Trinidad Meneses Caroca, Javiera Rojo-Gonzalez, Mathias Ignacio Orellana-Donoso, Pablo Nova-Baeza, Alejandra Suazo-Santibañez, Juan Sanchis-Gimeno, Hector Gutierrez-Espinoza

**Affiliations:** 1 Departament of Morphology, Faculty of Medicine, Universidad Andres Bello, Santiago 8370071, Chile; juan.kine.2015@gmail.com (J.J.V.-F.); fpeasantibez@uandresbello.edu (F.P.-S.); a.salinasvergara@uandresbello.edu (A.V.S.); t.menesescaroca@uandresbello.edu (T.M.C.); j.rojogonzlez@uandresbello.edu (J.R.-G.); pablo.nova@usach.cl (P.N.-B.); 2 Departamento de Ciencias Química y Biológicas, Facultad de Ciencias de la Salud, Universidad Bernardo O’Higgins, Santiago 8370993, Chile; 3 Escuela de Medicina, Universidad Finis Terrae, Santiago 7501015, Chile; mathor94@gmail.com; 4 Department of Morphological Sciences, Faculty of Medicine and Science, Universidad San Sebastián, Santiago 7510157, Chile; 5 Faculty of Health Sciences, Universidad de las Américas, Santiago 8320000, Chile; a.suazo@uandresbello.edu; 6 GIAVAL Research Group, Department of Anatomy and Human Embryology, Faculty of Medicine, University of Valencia, 46001 Valencia, Spain; juan.sanchis@uv.es; 7 One Health Research Group, Universidad de las Américas, Quito 170124, Ecuador

**Keywords:** hepatic hilum, liver hilum, clinical anatomy, anatomical variants

## Abstract

**Background:** The liver has a region called the hepatic hilum (HH) where structures enter and exit: anteriorly, the left and right hepatic ducts; posteriorly, the portal vein; and between these, the left and right hepatic arteries. The objective of this review is to know how variants in structures of the hepatic hilum are associated with clinical alterations of the liver. **Methods:** The databases Medline, Scopus, Web of Science, Google Scholar, CINAHL, and LILACS were researched until January 2024. The methodological quality was evaluated with an assurance tool for anatomical studies (AQUA). The pooled prevalence was estimated using a random effects model. **Results:** A total of six studies met the selection criteria established in this study for meta-analysis. The prevalence of hepatic hilus variants was 9% (CI = 5% to 13%), and the heterogeneity was 83%. The other studies were analyzed descriptively and with their respective clinical considerations in the presence of the variant, such as the high incidence of the Michels type III variant; among the portal vein variants, the type III variant of the Cheng classification stands out and in biliary anatomy, and the IIIa variant stands out according to the Choi classification. **Conclusions**: This review allowed us to know in detail the anatomical variants of HH; the structure with which the greatest care should be taken is the hepatic artery because of the probability of metastatic processes due to increased blood distribution in the hepatic lobules. Finally, we believe that new anatomical and clinical studies are needed to improve our knowledge of the relationship between HH variants and liver alterations or surgeries.

## 1. Introduction

With respect to the literature on the anatomical variations of the structures that make up the hepatic hilum, there are few systematic reviews that group and characterize them as a whole. Therefore, the aim of this systematic review is to analyze the characteristics and clinical implications of anatomical variations of the hepatic hilum worldwide in the 21st century.

**Figure 1:**
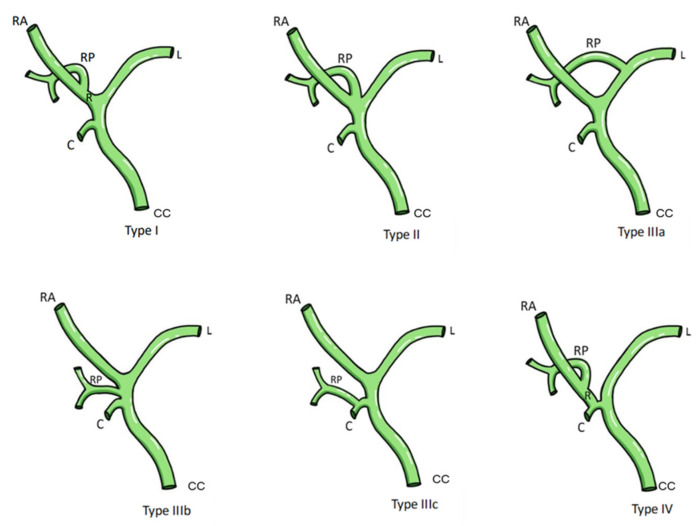
Classification biliary tree anatomy variation according to Kirimker et al. [2]. Abbreviations: RA: right anterior; RP: right posterior; L: left; C: cystic; R: right; CC: common hepatic duct.

**Figure 2:**
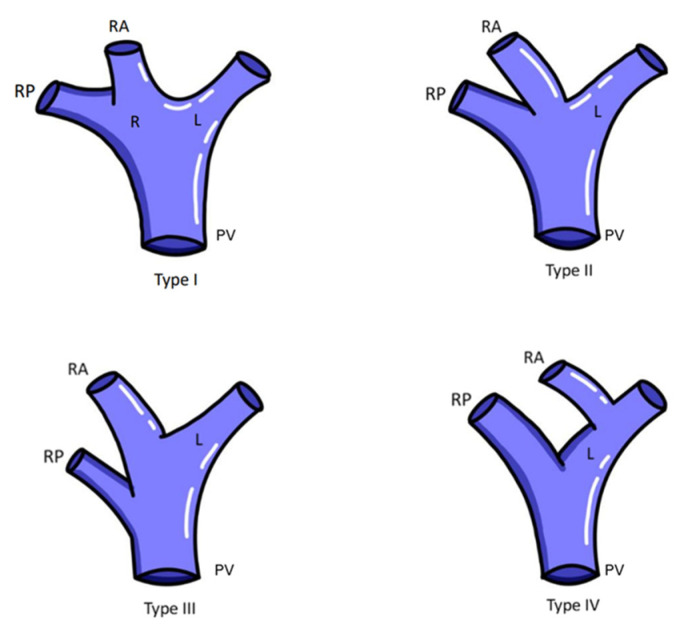
Classification of portal vein anatomy variation according to Cheng et al. [3]. Abbreviations: RA: right anterior; RP: right posterior; L: left; R: right; PV: portal vein.

**Figure 3:**
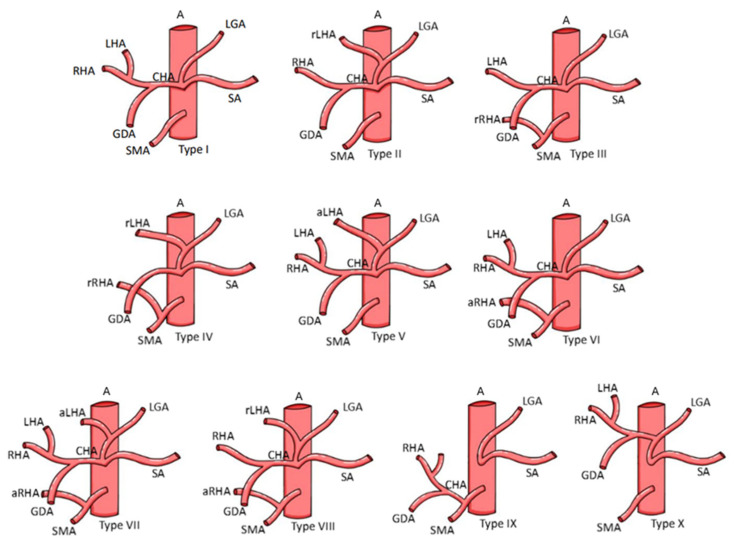
Classification of hepatic artery anatomy variation according to Michels [4]. Abbreviations: LHA: left hepatic artery; aLHA: accessory left hepatic artery; CHA: common hepatic artery; RHA: right hepatic artery; LGA: left gastric artery; SA: splenic artery; SMA: superior mesenteric artery; GDA: gastroduodenal artery; aRHA: accessory right hepatic artery; A: aorta.

The liver is a large organ that is divided externally into four lobes: two main and two accessory. This due to the reflections that the peritoneum forms on its surface of the fissures and the vessels that irrigate the liver. The four lobes are divided into eight hepatic segments by the transverse passage of the hepatic branches of the portal triad. Each segment receives a tertiary branch of the portal triad; in addition, each segment has its own intersegmental vascularization and biliary drainage. The liver has a region called the hepatic hilum where structures enter and exit: anteriorly, the left and right hepatic duct; posteriorly, the portal vein; and between these, the left and right hepatic artery [1]. Regarding the classifications of hepatic hilum (HH) variants, the Choi’s classification reported in Krimker et al. [2] is proposed for the variations of the hepatic ducts (Table 1 and Figure 1), the classification in Cheng et al. is proposed for the variants of the hepatic portal vein [3] (Table 2 and Figure 2), and the classification in Michels [4] is proposed for hepatic artery variants (Table 3 and Figure 3).

The distance of the left bile duct and the other bile ducts described by Kawarada et al. [5], whose midsegment variation was described as a type I variation, occurred in 35% of the 141 subjects. When analyzing studies of over 20 individuals, Lee et al. [6] showed three different types of bile ducts attached to the left hepatic duct.

The right anterior portal vein variation with RLDRH presented in 11.8% [6]. Along this same line, Ozsoy et al. [7] presented a study in which classified variations of the portal vein were observed, identifying type I in 78.6% of donors. The typical anatomy of the right hepatic duct [8], examined in a total of 73 subjects, showed a right posterior duct with fusion in 11 of them. Michaels [9] investigated the celiac trunk; 55% were normal, and 25% showed abnormalities. In the study, 10 types of irrigation variants were discovered; 55% were variant 1, and 10% were variant 2.

Hepatic artery anatomy is subdivided into 10 types, described by Michels [4], who found variant 1 in 144 patients [4]. In Italy, an investigation evaluated 40 volunteers, of whom 26 had significant variation in the transbiliary division [9]. In Germany, Radtke et al. [10] conducted a study of 165 donors, in which 40.7% had a star-shaped portal vein branching pattern. In Türkiye, 386 patients were evaluated for variations of the portal vein, with type II observed in 20 patients with trifurcation [11]. Years earlier, Yaprak et al. [12] performed a study of 200 donors, evaluating the anatomy of the hepatic artery in a classical manner. Fifteen percent had portal vein variation, and 63% had normal biliary anatomy [12].

In China, a study of 97 patients showed a normal biliary confluence pattern in 53. The left hilar system (type I) was present in 69 patients. The right hilar system of the investigation was based on the presence or absence of the hepatic duct, which was absent in 22 patients [13]. Another study by Wang et al. [14] evaluated 145 patients, where 103 had AHM.

Multiple studies were performed in Korea. One of them analyzed 300 donors, classifying seven variants of the hepatic duct; type I was found in 188 cases [15]. Another by Yu et al. [16] analyzed 64 cadavers presenting portal vein variations; type I, found in 228 patients, was the most common. Another study of 33 patients related variations in the length of the bile duct [17]. Finally, a study of 43 patients analyzed the anatomy of the hepatic artery according to Michels [4]. Type I was present in 10/11, and a candidate for type V was highlighted [18]. In Japan, a study was performed on 67 mixed patients. Portal vein branching was observed, with 60 cases of type P-I, the most common type. As for the hepatic artery, type A-I was observed in 39 cases, and the type B-I bile duct variant was seen in 53 [19].

However, in each study, different variations are described, but they are related to the anatomy or composition of the hepatic hilum. Therefore, the exact prevalence of these anatomical variants is not described in the literature.

## 2. Methodology

### 2.1. Protocol and Registration

This study was conducted and reported according to the PRISMA statement guidelines [20]. Its registration number in the International Prospective Register of Systematic Reviews (PROSPERO) is CRD42024507808.

### 2.2. Electronic Search

We systematically searched MEDLINE (via PubMed), Google Scholar, Web of Science (WoS), Cumulative Index to Nursing and Allied Health Literature (CINAHL), Latin American and the Caribbean Literature in Health Sciences (LILACS), and Scopus databases from inception to 31 January 2024. Our search strategy included a combination of the terms “hepatic hilum” (Not MeSH), “liver hilum” (Not MeSH), “clinical anatomy” (Not MeSH), and “anatomical variation” (Not MeSH), using the Boolean connectors AND, OR, and NOT (Supplementary Table S1).

### 2.3. Eligibility Criteria

Studies that covered the presence of HH variants and their association with a clinical condition were included. They were considered eligible for inclusion if the following criteria were met: (1) for samples, dissections or images with the presence of the HH variation; (2) for results, the prevalence of subjects who presented HH variants and their correlation with pathologies of the supramesocolic region; and (3) for studies, research articles and retrospective and prospective observational types, published in English in peer-reviewed journals and indexed in the reviewed databases.

Regarding exclusion criteria, we eliminated the following from our selection: (1) for samples, studies carried out in animals; (2) studies that analyzed variants of the region or system outside the hepatic region or its drainage area or tract; and (3) for studies, letters to the editor or comments.

### 2.4. Selection of Studies

To make a thorough selection of studies, three authors analyzed them independently. First, two authors (F.P. and A.V.) examined the titles and abstracts of the references recovered from the database searches. For the selected studies, the full text of the references that any of the authors considered potentially relevant was obtained. A third researcher (T.M.) was involved if a consensus could not be reached. For this purpose, we have also performed the agreement test between authors. Two authors analyzed the number of total studies after the search filter and eliminated all duplicates to see the degree of agreement in the selection. In total, 56 full-text articles were analyzed. The first reviewer (J.V.) selected 26 studies, and the second author (F.P.) selected 18. After this analysis, the coincidence was 20 studies, giving a Kappa index of 0.88.

### 2.5. Data Collection Process

Two authors (J.R. and J.J.V.) independently extracted data on the outcomes of each study. The following data were extracted from the included studies: (a) authors and year of publication, (b) total N and age, (c) prevalence, (d) characteristics of variant, (e) geographical region, (f) sex, and (g) clinical considerations.

### 2.6. Assessment of the Methodological Quality of the Included Studies

To evaluate the bias in the included studies, we used the verification table for anatomical studies (AQUA) proposed by the International Working Group on Evidence-Based Anatomy (IEBA) [21]. Two researchers (J.J.V. and P.N.) independently analyzed the 5 domains proposed by the AQUA tool and then reached a consensus and constructed the table and the bias graph.

### 2.7. Publication Bias

Using JAMOVI statistical software (version 14.1.2), we constructed a funnel plot. For publication bias, we have the funnel plot graph, where theoretically the data that most affected this criterion were the statistical significance of the primary article and its sample. This graph crossed the sample measurement against the exposure association or confidence interval transformed into standard error against the sample size.

### 2.8. Statistical Methods

For the statistical analysis, we used the JAMOVI technological tool. We included the data in a binary way and continuously to obtain the proportion we have expressed in prevalence; the statistical model used to combine the summarized data was the DerSimonian–Laird with a Freeman–Tukey double arcsine transformation. Additionally, a random effects model was used because the VD prevalence data were very heterogeneous. The degree of heterogeneity among the included studies was assessed using the Chi^2^ test and the heterogeneity statistic (I^2^). Finally, with the JAMOVI tool, we analyzed a funnel plot graph representing the magnitude of the measured effect.

## 3. Results

**Figure 4:**
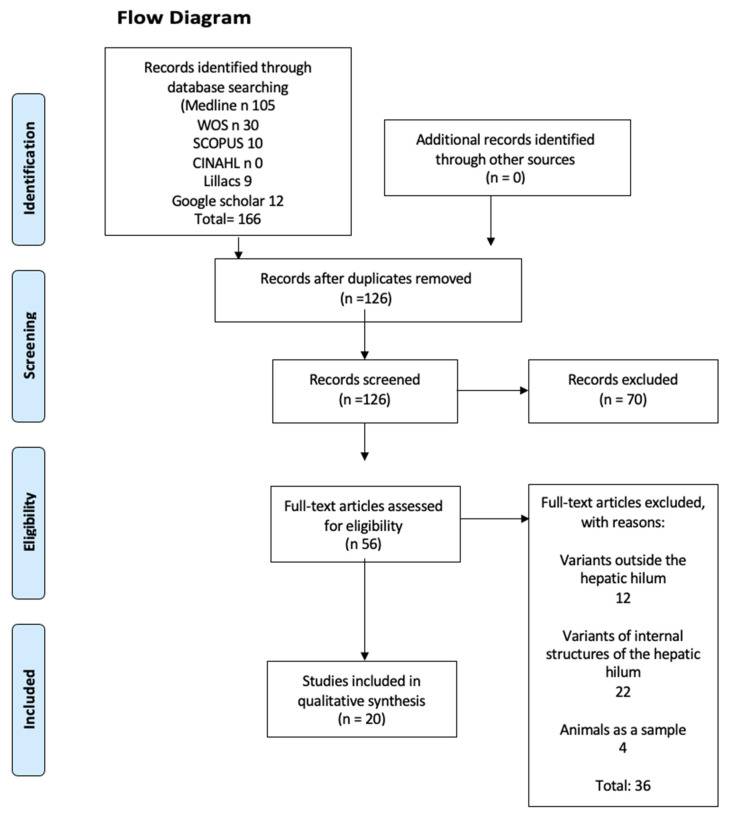
Flow diagram.

After performing the search and applying the inclusion and exclusion criteria, we added 20 studies, of which 18 were observational studies [2,4,5,6,7,8,9,10,11,12,13,14,15,17,18,19,22,23] and 2 were case studies [16,24] (Figure 4). Among the 20 included studies, 1 came from North America, 7 from Europe, and 12 from Asia, with a notable presence of the South Korean population. There were no studies from Oceania, Africa, or South America.

We included case studies because some of them provided us with information on anatomical variants rarely described in the literature, and some showed clinical considerations not frequently analyzed.

In some cases, the included studies distinguished between sexes. However, when studying the anatomical variations of the hepatic hilum, they did not differ between the two, eliminating the potential for bias.

In these 20 articles, a total of 2980 individuals were analyzed, coming mainly from Europe and Asia, with 1135 (39.77%) and 1519 (53.22%) subjects, respectively.

Of the sample of 2980 subjects, 1225 were male, 883 were female, and sex was not specified for 776 subjects. This is equivalent to 42.92% male, 30.94% female, and 27.19% unreported (Table 4).

### 3.1. Anatomy of Variants

Different variants of the HH components have been observed. In the hepatic artery, Michels’ type III variant has a high incidence; the common hepatic artery arises from the celiac trunk at the level of the T12 vertebra with the splenic artery and the left gastric artery. At the level of the omental orifice, the common hepatic artery bifurcates into the gastroduodenal artery and the proper hepatic artery, which in this case, in relation to the HH, originates a left proper hepatic branch but not a right one. An accessory or replacement right hepatic artery originates ectopically as a branch of the superior mesenteric artery; this can increase the diameter of the right proper hepatic artery branch, causing a space conflict or alterations in the shape of the HH. Among the variations of the portal vein, Cheng’s type III variant stands out. Normally, there is a division of the portal vein into a left, middle, and right branch; the right branch is divided into an anterior branch draining lobes V and VIII and a posterior branch draining lobes VI and VII. The type III variation implies an early segmentation of the right posterior branch of the portal vein, which could affect its entrance to the HH or cause an increase or decrease in the volume of these branches, altering the normal anatomy or arrangement of the structures of the HH. In the biliary anatomy, a variant that stands out in Kirimker et al.’s [3] classification is the type IIIa variation, which consists of the anomalous drainage of the right posterior segmental duct, which flows into the left hepatic duct and not into the right hepatic duct. As with the previously mentioned variants, this could cause a problem of space or the arrangement of structures in the HH.

### 3.2. Prevalence and Risk of Bias

**Figure 5:**
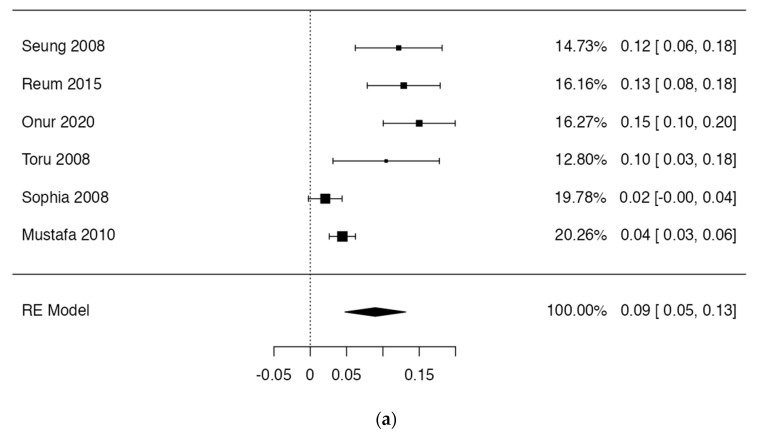

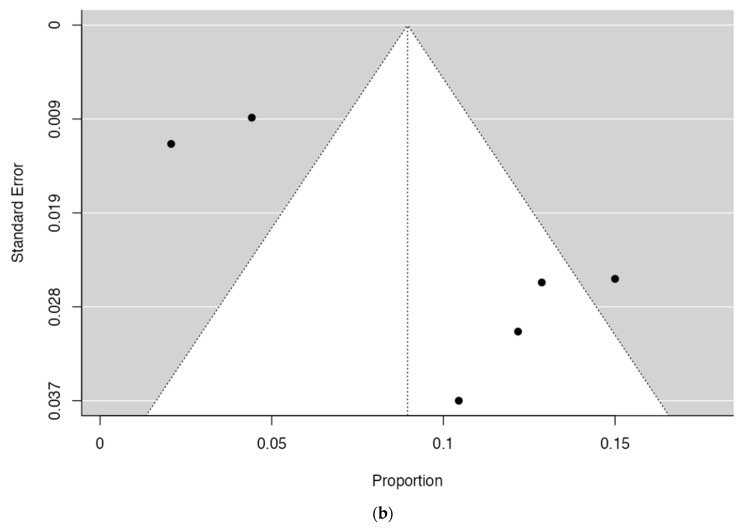
(**a**) Forest plot of prevalence. (**b**) Funnel plot of prevalence.

**Figure 6:**
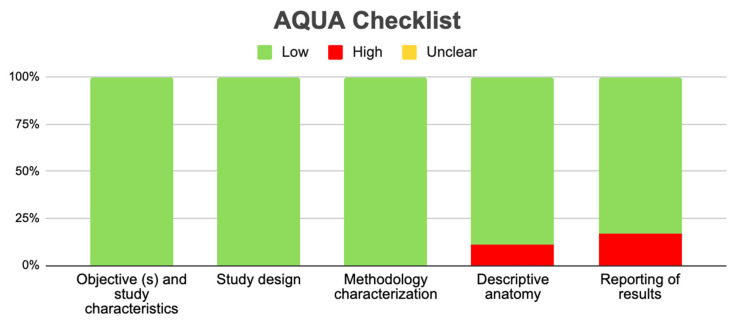
Risk of bias studies included with AQUA tools.

Regarding the prevalence of HH variants, six studies were included that satisfied the criterion that the individual prevalence of each study should not exceed 15% [2,6,7,19,22]. The presence of HH variants was 9%, with a confidence interval of 5–13% and heterogeneity of 83% (Figure 5). The funnel plot showed an asymmetry among the included studies, which indicated a high publication bias among the studies included in the meta-analysis (Figure 6). In conclusion, the 18 observational studies included in this review and analyzed with the AQUA bias tool presented a low risk of bias in the first three domains for all included studies; in domain 4, two studies showed a high risk of bias [2,22], and in domain 5, three studies showed a high risk of bias [2,4,15] (Figure 6 and Supplementary Table S2).

### 3.3. Clinical Considerations

Partial liver resection for living donor transplantation and the treatment of liver tumors is an important task. It requires detailed knowledge of the structural and vascular anatomy of the region to be treated, with emphasis on the HH anatomy, for safe and successful results.

Liver transplantation involves removing part of the liver from the living donor in such a way that it does not endanger its vascular supply or metabolic function. An imagenological evaluation is always suggested, to determine the donor’s compatibility and the contraindications for graft donation. This is also a crucial moment to identify anatomical variants that may alter the surgical approach.

The clinical implications of the variants of a structure such as the portal vein are significant in the management of various liver treatments. There is an isolated variant of the hepatic portal vein system, where the main portal vein does not generate branches and communicates directly in a sinuous way with the hepatic vein. If this is present in the imagenologic diagnosis of the living donor or in the recipient, it is a contraindication for right lobe hepatic transplants, rendering the surgical procedure unfeasible due to a transplant incompatibility [25].

Complications may arise in living donor transplantation if a variant is associated with either of the two right branches of the portal vein, which are structurally arranged one anteriorly and the other posteriorly within the hepatic lobes. For example, if the right posterior branch originates at the level of the portal vein trunk, or if the portal vein branching from the right anterior or posterior trunk has a leftward disposition, transplantation may be contraindicated due to the risk to the living donor from incompatibility with the recipient.

Another scenario to consider is if the trifurcation is not correctly detected. In this case, if the middle branch of the hepatic portal vein, which corresponds to one of the two right branches, is sectioned, the vascularization of the anterior or posterior segments of the donated right lobe is put at risk, which could cause a loss of tissue or alterations in the vascularization of the region where the variant is located.

If the portal vein variants occur after lobar segmentation and present a correct disposition toward each lobe, the contraindications will be fewer, or the transplantation may even perform more favorably in the donor.

In the biliary tract, different variants are observed, which can present complications for living donors, as well as for recipients in liver transplant surgeries. These include the ones that are connected to the separation of the ducts of the different liver segments, such as the left hepatic duct joined to segment IV, two separate ducts of the right lobe branching before the cystic duct, the exit in the hepatic duct of segment IV toward the right and left hepatic duct, the trifurcation of the biliary ducts in segment II and III, and a duct of segments V and VIII that branched from the left hepatic duct just behind the point where the hepatic ducts of segments VI and VII and the left hepatic duct join. If the disposition of these variants is proximal to or closely associated with the right lobe, they will generate a greater probability of incompatibility between the living donor and the recipient [26].

In the case of finding a carcinoma in the region of the hepatic hilum, the biliary ducts must be taken into account to evaluate the hepatic resection to be performed since, for example, if B4 is near B11 (Figure 5), the resection must be of both S4a and S1 and the extrahepatic biliary tract must be removed (Figure 5a,b). On the contrary, if B4 is distant from B11 (Figure 6), removal of S4a will not be necessary, since invasion into B4 is rare in these cases. This demonstrates the importance of knowing the anatomy of the bile ducts and their possible variants [5]. Additionally, the anatomy of the hepatic arteries must be known since as mentioned by Avila [27], tumors normally seek accessory pathways in order to receive blood supply; this distribution must be preserved to avoid any hepatobiliary and pancreatic surgical complications.

The section of a variant of the hepatic artery can lead to necrosis of one or more hepatic segments; for example, in the case described by Avila [27], an accessory hepatic artery of the left hepatic artery is found that does not re-anastomose the left hepatic artery, since it could cause damage to the right hepatic artery. Therefore, the greater the accessory irrigation, which corresponds to anatomical arterial variants that will cause an increase in the irrigation of this tumor, the greater the risk of metastasis and in the surgical procedure since all these extra variants caused by the resection of the tumor must be evaluated.

It is important to consider variations of the hepatic portal vein. There are procedures such as segmentomy, which consists of a complete resection of an area irrigated by a segmental branch of the portal vein, with the aim of preserving liver function because patients with hepatocellular carcinoma develop chronic hepatitis and cirrhosis [28].

When performing a left hepatectomy in a living related donor for transplantation, it is important to recognize aberrant drainage of the right posterior duct or right anterior duct into the left hepatic duct because ligation of these ducts will result in biliary cirrhosis of segments VI and VII, or segments V and VIII, respectively [8].

Finally, in the case of a cadaveric donor transplant, the risk factors and incompatibilities mentioned above are reduced, making the transplant safer and more feasible in the face of various eventualities.

## 4. Discussion

The purpose of this review was to understand the anatomy of the HH and the variants associated with the main components that entered and exited it. In addition to the prevalence with which changes occurred, we also studied how these variants were associated with clinical alterations in the liver. We found that the presence of variants of the HH alone did not produce symptoms or classic pathological signs but was clinically associated with the pathologies of surrounding areas or in certain surgical procedures.

We did not find any previous studies on anatomical variants of the HH that performed any clinical review associated with them, so this work is novel. However, we also understand that many studies report the variants of the hilum as specific studies of each structure, either hepatic portal vein, proper hepatic artery, or common hepatic ducts. Therefore, we conducted a search for previous reviews of the individual structures and found two reviews that analyzed the variants of the portal vein and its relationship with clinical considerations.

A review by Prado and Petroianu [29] reveals that HPV variants are predominantly associated with their origin. The existing body of literature covers the complexities of diversity in HPV formation, while our research focused specifically on elucidating variants entering the hepatic hilum and performing an in-depth investigation of rare variants; thus, a meticulous meta-analysis of these specific elements was performed. In contrast, the review by Stefura [30] highlights mainly HPV-related collateral variants. This review, however, does not delve into the discussion of variants in HPV formation or its entry into the hepatic hilum; it refrains from performing a meta-analysis on these specific variants.

For variants of the hepatic artery proper, Zhang [31] in their review showed surgical resection of multicentric hepatobiliary and pancreatic tumors in such rare SIT anatomic anomalies with vascular variants. The main variation mentioned is type lX, in which the common hepatic artery (CHA) arises from the superior mesenteric artery (SMA). So in these cases a reliable surgical plan based on detailed preoperative imaging and intraoperative anatomic exploration should be considered to achieve radical resection.

In the research of Jin Woo Choi [15], at least seven variations on intrahepatic bile ducts are presented; the most prevalent are type l, which is considered the typical structure, followed by the type 3a variation, which is the anomalous drainage of the right posterior segmental duct (RPSD) in the left hepatic duct. The study emphasizes the importance of knowing the anatomy of these variants to ensure a successful liver resection and decrease postoperative complications. This is especially critical in percutaneous biliary procedures since they can lead to drainage of the left side of the liver and the posterior segment of the right side.

In this study, although there were more males than females (excluding the studies in which sex was not reported), we believe that this variant is not related to the sex of the subject. The data may be associated with the collection of the sample and not any sex factor associated with the HH variants. Our study also included a high prevalence of Asian subjects, especially South Koreans, with variations of the bile ducts and Europeans, especially subjects from Türkiye, with variations of the hepatic arteries and portal vein. Finally, a minority of our subjects were from the United States, specifically from the state of Pennsylvania, reporting mostly variations in hepatic arteries in relation to the HH. Although most of the studies in this review were from two continents, we can make no certain association between HH variants and races from these continents; instead, we believe that the higher numbers can be attributed to a greater number of studies investigating these variants in these populations.

Concerning anatomy, we studied the three components that formed the HH. The variants that produced the greatest alteration in the formation of the HH were the following: a variant of the hepatic artery in which a right hepatic artery arises from the superior mesenteric artery, a variant of the portal vein that consists of an early segmentation of the right posterior branch of the portal vein, and a variant of the biliary duct where the right posterior segmental duct drains into the left hepatic duct. Medical teams and professionals who approach the hepatic region should be aware of these variants as they could be crucial in surgical management or in avoiding some associated differential diagnoses.

Of the studies included, less than 30% met the criteria to perform the prevalence meta-analysis as most chose their samples intentionally and included mainly subjects presenting the HH variant. The prevalence of this variants was 9%, which is high for an anatomical variant. We believe that these data could be overestimated and should be taken with caution. Additionally, there was a high risk of publication bias, which indicates that the studies were very heterogeneous. Therefore, more studies are needed to support this observation regarding the HH variant. There was a low risk of bias in the included studies, which indicates that they were methodologically well structured and that the studies were correctly chosen for this review.

For clinical considerations, it is important to know in detail the anatomical variants of the HH, such as in the case of living donor transplantation, tumor removal, or any partial or total resection of a hepatic segment. This knowledge allows for successful diagnoses and procedures and prevents complications.

Living donor liver transplantation consists of removing a part of the donor’s liver without compromising its blood supply or metabolic capacity [32]. Variants of the portal vein have important clinical implications and can lead to complications in living donor transplantation. For example, if the right posterior branch originates from the main trunk of the portal vein, transplantation is complicated or not feasible due to the risk to the donor, as there would be no match with the recipient. Similarly, a left-sided orientation of the anterior or posterior right branches of the portal vein could also result in incompatibility between the donor and recipient [33].

We found no studies reporting that HH variants produce any pathologies or signs in patients. The variant can be recognized through pathologies that affect surrounding structures, which means that this variant can be present throughout life without producing symptomatology on its own. Clinical considerations are very rarely associated directly with the structure surrounding the HH but present separately. The portal vein, within the structures of the HH, has great clinical importance in liver treatments and can invalidate surgical procedures due to incompatibility or increase the risk of pre- and post-surgical complications. These complications are generated mostly by the absence of branches of the portal vein or structural differences in the arrangement of the branches. If they are more intense or closer to the lobes, the risk for complications decreases.

Hepatocellular carcinoma can sometimes lead to chronic hepatitis or cirrhosis. In these cases, the feasibility of resection must be evaluated through procedures such as segmentomy. It must be recognized especially if the portal vein gives its ramifications proximally, which makes surgical reconstruction more complex [28].

In relation to cancer, the biliary ducts must be evaluated for any possible invasion of the tumor in the liver through the biliary tract. For transplants, the anatomy of the living donor and the recipient must be evaluated to determine the probability of incompatibility between them. In addition, it must be considered that certain ducts can be ligated with a specific hepatic segment. This ligation relationship with some segments can produce biliary cirrhosis, which must be considered and explained to the patient due to the possibility of complications [8].

In the case of cadaveric transplants, there is a significantly lower risk for rejection. Since the vessels are rearranged, new anastomoses can be generated, and a safer reconstruction of the HH can be achieved.

Finally, in relation to the hepatic arteries, tumors seek blood supply, so any anatomical variation around a tumor should be evaluated to appreciate its reception and possible complications, such as necrosis in the liver or other related organs. This is a greater concern if the hepatic artery itself originates from the superior mesenteric artery since it comes from another region of the abdominal aorta and not from the celiac trunk.

## 5. Limitations

The limitations of this review were the publication and authorship bias of the included studies. Studies with different results that were in non-indexed literature in the selected databases may have been excluded. The authors selected the articles in personal sessions and may not have carried out the most sensitive and specific search on the topic to be studied. All of this may result in the exclusion of cases not reported in the scientific community or in countries outside of the Asian and North American continents.

## 6. Conclusions

This review provided a detailed understanding of the anatomical variants of the HH, showing that variants will individually affect one or more components of the HH. The clinical presentation or complications that each structure causes depend on the type of variant present, not on the age or sex of the patients. Prevalence cannot be taken as a reference, since different studies were performed on different types of patients. This review shows that in some surgeries, such as liver transplantation through a living donor, all HH components will present complications for the living donor or the recipient in relation to the hepatic ducts, portal vein, and variations of the aortic artery. In the presence of neoplastic diseases, the greatest care should be taken with the hepatic artery due to the probability of metastatic processes resulting from increased blood distribution in the hepatic lobules. Finally, we believe that new anatomical and clinical studies are needed to improve our knowledge of the relationship between HH variants and liver alterations or surgeries that may lead to complications. Furthermore, increased knowledge of these variations through new imaging and surgical technologies would allow for greater precision in observation and care during various future interventions for liver transplants in living donors, including robotic hepatectomy [34].

## Figures and Tables

**Table 1:** Classification of biliary anatomy according to Kirimker et al. [2].

Type	Type 1	Type 2	Type 3	Type 3a	Type 3b	Type 3c	Type 4
Description	Typical	Triple confluence (right anterior segmental duct, right posterior segmental duct, and left hepatic duct).	Right posterior segmental canal drains abnormally.	Drains into the left hepatic duct.	Drains into the common hepatic duct.	Drains into the cystic duct.	Right hepatic duct drains into the cystic duct.

**Table 2:** Classification of the portal vein (PV) according to Cheng.

Type	Type 1	Type 2	Type 3	Type 4
Description	Single division of the left and right lobes of PV.	Trifurcation of the anteroposterior segments of the right PV with the left PV.	Early segmentation of the right posterior branch of PV.	Anterior sectoral branch of the umbilical portion of the left PV.

**Table 3:** Classification of the hepatic artery according to Michels [4].

**Type**	**Type 1**	**Type 2**	**Type 3**	**Type 4**	**Type 5**
Description	Right, left, and middle hepatic artery.	The right and middle hepatic arteries. Left hepatic artery replaced from left gastric artery.	The left and middle hepatic arteries. The right hepatic artery was replaced from the superior mesenteric artery.	The right hepatic artery was replaced from the superior mesenteric artery. The left hepatic artery replaced the left gastric artery.	The right, middle, and left hepatic arteries. The accessory left hepatic artery from the left gastric artery.
**Type**	**Type 6**	**Type 7**	**Type 8**	**Type 9**	**Type 10**
Description	The right, middle, and left hepatic arteries. An accessory right hepatic artery arising from the superior mesenteric artery.	An accessory right hepatic artery from the superior mesenteric artery and an accessory left hepatic artery from the left gastric artery.	Combined patterns of a replaced right hepatic artery and an accessory left hepatic artery or vice versa.	The entire hepatic trunk derives from the superior mesenteric artery.	The entire hepatic trunk derives from the left gastric artery.

**Table 4:** Characteristics of the included studies.

Author	Total N and Age	Prevalence	Characteristics of Variants	Geographic Region	Sex	Clinical Consideration	Type of Study	Techniques Used
Lapisatepun 2023	1 example and 45 years	1 (100%)	Not portal vein bifurcation	Thailand	Female	The explant portal vein bifurcation is a useful vascular graft for the reconstruction technique in this type of variation.	Case studies	Laparoscopic donor right hepatectomy
Kawarada 2000	141 (68 adult carcasses and 73 liver casts)	Type I: 50 Type II: 77 Mixed: 14 Average distance: Type I: 34 of 56 Type II: 22 of 56	According to the union of B4: Type I: near the hilar confluence Type II: away from the hilar confluence Mixed: uniting the near and the far Type I (8 mm) distance. Type II (17 mm) distance.	Japan	Not reported	The presence of a type 1 variant of the hepatic hilum was associated with bile duct carcinoma.	Observational	Dissections of cadaveric specimens and biopsy
Lee 2008	MRI scans of 115 people Mean age of 32 ± 9 years (range 18–53 years)	Type I: 101 Type II: 6 Type III: 8	Type III: B4 (bile duct) joins the left lateral section of the hepatic duct, lateral to the umbilical portion.	Seoul, South Korea	83 males 32 females	In 7% of cases, B4 was attached laterally to the umbilical portion, a situation in which B4 can be injured during left lateral sectionectomy.	Observational	Diagnostic imaging of donors
Kim 2022	171 Before 2019 RLDRH 102 age: 30.7 ± 9.4 LLDRH 69 age: 30.4 ± 10.6 After 2019 125 RLDRH age: 32.5 ± 9.6 LLDRH: 30.4 ± 10.6	Hepatic artery and portal vein variation was always greater in RLDRH than in LLDRH, before and after 2019.	Accessory hepatic artery. And 2 from portal vein, a tribifurcation And the right anterior portal vein from the left portal vein.	South Korea.	RLDRH and LLDRH Males: 80 Females: 91 After 2019: RLDRH and LLDRH Males: 56 Females: 69	Graft-related complications, such as vascular and biliary complications, were statistically similar in both.	Observational	Laparoscopic donor right hepatectomyr (LLDRH) and robotic donor right hemiheoatectomy (RLDRH)
Mariolis 2012	73 samples of cadaveric incisions	a. Typical anatomy: 48 (65.75%) b. 11 (15.07%) c. 7 (9.59%) d. 3 (4.11%) e. 2 (2.74%) f. 1 g. 1	b. Right posterior duct fused with the right or left anterior hepatic duct c. Triple confluence toward the common hepatic duct	Athens, Greece	35 males 38 females	Liver surgical procedures require extensive knowledge of intrahepatic and extrahepatic anatomical variations, to avoid possible complications and achieve the most effective result.	Observational	Dissections of cadaveric specimen
Kirimker 2022	203 patients Age: 32.3 ± 8.6	Biliary anatomy: Type I: 129 Type II: 12 Type IIIa: 38 Type IIIb: 14 Type IIIc: 2 Type IV: 1 Type V: 5 Type VI: 2 Hepatic artery anatomy: 1: 144 2: 16 3: 23 4: 4 5: 6 6: 4 7: 1 8: 2 9: 1 10: 2	Biliary anatomy: Type IIIa: right posterior segmental duct drains into the left hepatic duct Hepatic artery anatomy: Michels type description	Helsinki, Philandia	77 females 26 males	Donor anatomical variations are not important risk factors for worse recipient survival or donor morbidity.	Observational	Living donor liver transplantation
Michels 1966	200 corpses; no information on age range	1: 110 2: 20 3: 22 4: 2 5: 20 6: 14 7: 2 8: 4 9: 5 10: 1	3: Right hepatic artery substituted from the superior mesenteric artery	Philadelphia, Pennsylvania	Not reported	In hepatic lobectomy and partial heparectomy, knowledge of the anatomy is essential to preserve the vital arteries.	Observational	Hepatic lobectomy and partial hepatectomy
Choi 2003	300 donors Age: 16–60 years Average: 30 years	Types: 1: 188 2: 29 3a: 34 3b: 19 3c: 6 4: 1 5a: 8 5b: 8 6: 4 7: 3	3a: drainage of the RPSD into the left hepatic duct	Seoul, Korea	229 males 71 females	Knowledge of the anatomy of the intrahepatic bile ducts is essential to successfully remove the liver with the least number of postoperative complications.	Observational	Intraoperative cholangiograms
Radtke 2009	71 donors Mean age: 37 ± 10.1 years	Incidence of anatomical anomaly: portal vein (11.3%), biliary duct (22.5%), hepatic artery (36.6%). Hepatic artery (36.6%) and in segmental vascular and biliary anatomy: portal vein (38%), biliary duct (46.5%), hepatic artery (25.4%)	Star-shaped branching pattern in portal vein Crossed segmental branching of the hepatic artery	Mainz, Germany	36 females 35 males	Accurate imaging cannot prevent complications due to unfavorable central hilar branching at the medial border of the right “hilar corridor”, as it is of “inherent origin”.	Observational	Living donor liver transplantation
Carollo 2021	40 volunteers; no information on age range	23 single artery and bile duct. In the rest, the number of elements varies.	Biliary drainage anatomy was normal, except in two cases. The left bile duct is a small one that runs vertically from right to left below the REX recess.	Palermo, Italy	Not reported	TH and TU helped to deal with the anatomical biliary problem of the patients and reduce the risk of biliary problems.	Observational	Transection trashiliar (TH) or transumbilical (TU) parenchymal trasnsection
Guler 2013	In total, 386 donors, 52 clinical data for portal vein variations; no information on age range	Portal vein anatomy was classified as follows: Type 2: 20 patients Type 3: 24 patients Type 4: 8 patients	Type 3: Early segmentation of the right posterior branch of the portal vein	Türkiye	Not reported	LDLT is a valuable treatment in the adult population but technically challenging due to high rates of vascular and biliary variations that can result in donor rejection.	Observational	Living donor liver transplantation (LDLT)
Hai 2017	78-year-old male	A male in the studio	Portal branch of the right lateral sector first diverged only from the main portal vein, and the right umbilical position branched into several portal branches to the left and right.	Japan	1 male	The vascular and biliary anatomy in patients with the right round ligament is complicated and must be evaluated in a hepatectomy system.	Observational	Diagnostic imaging of donor
Wang 2010	145 patients Age range 19–65 years	103 of the patients had MHA (71%). 5 types of MHA: MHA type I accounted for 43.7% (n = 45). MHA type II accounted for 26.2% (n = 27). MHA type III accounted for 12.6% (n = 13). MHA type IV accounted for 10.7% (n = 11). MHA type V accounted for 6.8% (n = 7).	MHA type 1: an MHA that origi- nated from an RHA in patients with a normal hepatic arterial configuration	Shenzhen, China	92 males 53 females	Type II MHA originating from RHA requires a more complex surgical technique than when MHA is absent. Ligation of MHA can lead to biliary complications in a left lobe allograft.	Observational	Diagnostic imaging of donors
Yaprak 2020	200 living liver transplant donors Males aged 37.4 (range 18–63) and females aged 50.7 (range 17–72)	Classical anatomy of the hepatic artery (64.5%) Variation of the portal vein (15%) Variation of the portal vein and biliary tract (70%) Variation in the biliary duct (28%)	Type 3; right hepatic artery substituted from the superior mesenteric artery. In the variations of the portal vein (5.5%) had a right anterior sectoral branch originating from the left portal vein.	Istanbul, Türkiye	118 males 82 females	The success of the transplantation procedure depends carefully on anatomical variations.	Observational	Diagnostic imaging of donors and living donor liver transplantation
Han 2014	33 patients out of 159 with Bismuth type IV hilar cholangiocarcinoma Age between 24 and 79 years old	In patients with CIR the biliary A2 variants are higher than in patients without CIR. Not found in patients with variations in portal vein and hepatic artery.	Variations of the bile ducts Type A2: hilar trifurcation at the common junction of the right anterior hepatic duct, right posterior hepatic duct, and left	Korea	Not reported	There was a survival gain in patients receiving CRT compared with patients who did not receive it. The long-term survival of patients with hilar cholangiocarcinoma is negative surgical settings, lymph node status, and tumor differentiation degree.	Observational	Major hepato–biliary resection
Ikegami 2008	67 between 20 and 60 years old	Prevalences were varied, the most typical being as follows: Branch of the portal vein, 60 cases, type P-I. Arterial branching/alteration, Type A-I: 39 Biliary: Type B-I: 53.	Branch of the portal vein, posterior branch (type P-I to P-III). Type A-I to A-III branching pattern of the hepatic artery. Bile duct according to tributaries of the posterior hepatic duct type B-I and B-IV.	Japan.	26 males 41 females	Hilar anatomic variations in liver transplants in living right lobe patients are not uncommon, and are manageable after detailed preoperative evaluation of the graft and appropriate intraoperative surgical technical decisions.	Observational	Living donor liver transplantation
Ji 2017	97 patients. No information on age range.	Left hilar system: Type I:69 (69.1%) Right biliary system: Absent right hepatic duct: 22 (22.7%), Right posterior bile duct 13 cases.	Left biliary system associated with the relationship of the hepatic segmental ducts Right biliary system according to the presence or absence of the corresponding hepatic duct Type I: Normal	China	61 males 36 females	Hemihepatectomy could be selected for resection with curative intent of BC type IV tumors, with the pattern of bile duct confluence considered advantageous.	Observational	Diagnostic imaging of donors
Yu 2011	Adult cadavers (n = 64) Tomographies (MDCT) of human livers (n = 216) No mention of age	Variation of PV: Type 1: 83% Type 2: 8% Type 3: 9% According to the form of ramifications, they were classified into 4 types: Type I: 121 (43.2%) Type Y: 145 (51.8%) Type V: 9 (3.2%) Type U: 5 (1.8%)	Type 1 originates from the PST posterior sectoral trunk. Type 2 originates from the confluence of PST and the lateral sectoral trunk Type 3 originates from the lateral sectoral trunk LST. RSRL the location of the gallbladder in these two cases was on the left side of the round ligament; they considered it as type 3. Branching types (I, Y, V, and U) not described.	Jeonju, Korea	Not reported	To avoid confusion resulting from branching order, they prefered to use horticultural terms such as trunk and branch instead of second and third order branches.	Case studies	Dissections of cadaveric specimens and diagnostic imaging of donors
Lim 2005	43 potential living liver donors. Age range, 16 to 52 years; mean age, 31.6 years.	There were many variations, but the most typical was the origin of the middle hepatic artery in the left hepatic artery, presented in 9 patients.	Middle hepatic artery: origin from the left hepatic artery Hepatic venous anatomy: accessory hepatic veins	Seoul, South Korea	13 females 30 males	This article does not establish a relationship between anatomical variants of the hepatic hilum and clinical implications.	Observational	Diagnostic imaging of donors
Ozsoy 2011	496 liver donors. Ages range between 18 and 64 years.	There were many variations, but the most typical was the left and middle hepatic vein draining into the IVC in a single orifice, in 315 patients.	Portal vein: Type 2: trifurcation. Middle and left hepatic veins drained into the IVC, joining into a single orifice.	Manisa, Türkiye	253 males 243 females	It is very important to know the possible variants of the hepatic artery and hepatic venous system to avoid damage to the donor during surgery.	Observational	Living donor liver transplantation

## Data Availability

No new data were created or analyzed in this study.

## Supplementary Materials

The following supporting information can be downloaded at https://www.mdpi.com/article/10.3390/life14101301/s1: Table S1: Searches for strategies. Table S2: Aqua checklist.

## Abbreviations

MRI	Magnetic resonance imaging
B4	Medial segment
RLDRH	Robotic living donor right hemihepatectomy
LLDRH	Laparoscopic living donor right hemihepatectomy
RPSD	Right posterior segmental posterior duct
TH	Trans-hilar division technique
TU	Trans-umbilical division technique
LDLT	Living donor liver transplantation
MHA	Middle hepatic artery
RHA	Right hepatic artery
CIR	Resection with curative intent
A2	Hilar trifurcation type
A	Aorta
CRT	Refused chemoradiotherapy
BC	Bismuth-Corlette
MDCT	Multi-detector computed tomography
PV	Portal vein
PST	Posterior sectoral trunk
LST	Lateral sectoral trunk
RSRL	Right-sided round ligament
IVC	Vena cava inferior
