# Supplementary material for: Hepatic Hilum Variations and Their Clinical Considerations in the Liver: A Systematic Review and Meta-Analysis
Source: Life (Basel). 2024 Oct 14;14(10):1301. doi: 10.3390/life14101301 (PMC11509691; doi:10.3390/life14101301)
Supplement: Supplementary file 1 [file life-14-01301-s001.zip › life-3041026-supplementary.pdf]

Supplemental Digital Content

Table S1. Searches for strategies.

**Database**	**Search strategy**	**Results**	
		**20-03-24**	
Medline	((("hepatic"[All Fields] OR "hepatophyta"[MeSH Terms] OR "hepatophyta"[All Fields] OR "hepatics"[All Fields]) AND "hilum"[All Fields]) OR (("hepatic"[All Fields] OR "hepatophyta"[MeSH Terms] OR "hepatophyta"[All Fields] OR "hepatics"[All Fields]) AND "hilium"[All Fields] AND ("variation"[All Fields] OR "variations"[All Fields]))) AND ("clin anat"[Journal] OR ("clinical"[All Fields] AND "anatomy"[All Fields]) OR "clinical anatomy"[All Fields]) AND (("liver"[MeSH Terms] OR "liver"[All Fields] OR "livers"[All Fields] OR "liver s"[All Fields]) AND ("anatomy and histology"[MeSH Subheading] OR ("anatomy"[All Fields] AND "histology"[All Fields]) OR "anatomy and histology"[All Fields] OR "anatomy"[All Fields] OR "anatomy"[MeSH Terms] OR "anatomies"[All Fields])) AND (("hepatic"[All Fields] OR "hepatophyta"[MeSH Terms] OR "hepatophyta"[All Fields] OR "hepatics"[All Fields]) AND ("anatomy and histology"[MeSH Subheading] OR ("anatomy"[All Fields] AND "histology"[All Fields]) OR "anatomy and histology"[All Fields] OR "anatomy"[All Fields] OR "anatomy"[MeSH Terms] OR "anatomies"[All Fields]))	105	
SCOPUS	hepatic hilum OR hepatic hilium variations AND clinical anatomy AND liver anatomy AND hepatic anatomy	10	
Lilacs	hepatic hilum OR hepatic hilium variations AND clinical anatomy AND liver anatomy AND hepatic anatomy	9	
CINHAL	hepatic hilum OR hepatic hilium variations AND clinical anatomy AND liver anatomy AND hepatic anatomy	0	
WOS	hepatic hilum OR hepatic hilium variations AND clinical anatomy AND liver anatomy AND hepatic anatomy	30	
Google scholar	hepatic hilum OR hepatic hilium variations AND clinical anatomy AND liver anatomy AND hepatic anatomy	12	
	Total	166	

* All searches were carried out on March 20, 2024.

Table S2. Aqua checklist.

References	Study design	Domain 1				Domain 2					Domain 3						Domain 4					Domain 5				
		**1**	**2**	**3**	**4**	**5**	**6**	**7**	**8**	**9**	**10**	**11**	**12**	**13**	**14**	**15**	**16**	**17**	**18**	**19**	**20**	**21**	**22**	**23**	**24**	**25**
Lapisatepum et al., 2023 [24]	**Estudio de caso**	**Y**	**Y**	**Y**	**N**	**Y**	**Y**	**Y**	**N**	**Y**	**N**	**Y**	**Y**	**Y**	**Y**	**Y**	**N**	**Y**	**Y**	**N**	**N**	**Y**	**Y**	**Y**	**Y**	**N**
Kawarada et al., 2000 [5]	**Estudio observacional**	**Y**	**Y**	**Y**	**N**	**Y**	**Y**	**Y**	**N**	**Y**	**Y**	**Y**	**Y**	**Y**	**Y**	**Y**	**N**	**Y**	**Y**	**N**	**N**	**Y**	**Y**	**N**	**Y**	**N**
Lee et al., 2008 [6]	**Estudio observacional**	**Y**	**Y**	**Y**	**N**	**Y**	**Y**	**Y**	**N**	**Y**	**N**	**Y**	**Y**	**Y**	**Y**	**N**	**N**	**Y**	**Y**	**N**	**N**	**Y**	**N**	**Y**	**Y**	**N**
Kim et al., 2022 [22]	**Estudio observacional**	**Y**	**Y**	**Y**	**N**	**Y**	**Y**	**Y**	**N**	**Y**	**Y**	**Y**	**Y**	**Y**	**Y**	**Y**	**N**	**Y**	**Y**	**N**	**N**	**Y**	**Y**	**N**	**Y**	**Y**
Mariolis et al., 2012 [8]	**Estudio observacional**	**Y**	**Y**	**Y**	**N**	**Y**	**Y**	**Y**	**N**	**Y**	**Y**	**Y**	**Y**	**Y**	**Y**	**Y**	**N**	**Y**	**Y**	**N**	**N**	**B**	**Y**	**Y**	**Y**	**N**
Kirimker et al., 2022 [2]	**Estudio observacional**	**Y**	**Y**	**Y**	**N**	**Y**	**Y**	**Y**	**N**	**Y**	**N**	**Y**	**Y**	**Y**	**Y**	**Y**	**N**	**Y**	**Y**	**N**	**N**	**Y**	**Y**	**Y**	**Y**	**N**
Michels 1966 [4]	**Estudio observacional**	**Y**	**Y**	**Y**	**N**	**Y**	**Y**	**Y**	**N**	**Y**	**Y**	**Y**	**Y**	**Y**	**Y**	**Y**	**N**	**Y**	**Y**	**N**	**N**	**Y**	**Y**	**N**	**Y**	**N**
Choi et al., 2003 [15]	**Estudio observacional**	**Y**	**Y**	**Y**	**N**	**Y**	**Y**	**Y**	**N**	**Y**	**N**	**Y**	**Y**	**Y**	**Y**	**N**	**N**	**Y**	**Y**	**N**	**N**	**Y**	**N**	**Y**	**Y**	**N**
Radtke et al., 2009 [10]	**Estudio observacional**	**Y**	**Y**	**Y**	**N**	**Y**	**Y**	**Y**	**N**	**Y**	**Y**	**Y**	**Y**	**Y**	**Y**	**Y**	**N**	**Y**	**Y**	**N**	**N**	**Y**	**Y**	**N**	**Y**	**Y**
Carollo et al., 2021 [9]	**Estudio observacional**	**Y**	**Y**	**Y**	**N**	**Y**	**Y**	**Y**	**N**	**Y**	**Y**	**Y**	**Y**	**Y**	**Y**	**Y**	**N**	**Y**	**Y**	**N**	**N**	**B**	**Y**	**Y**	**Y**	**N**
Guler et al., 2013 [11]	**Estudio observacional**	**Y**	**Y**	**Y**	**N**	**Y**	**Y**	**Y**	**N**	**Y**	**N**	**Y**	**Y**	**Y**	**Y**	**Y**	**N**	**Y**	**Y**	**N**	**N**	**Y**	**Y**	**Y**	**Y**	**N**
Hai et al., 2017 [23]	**Estudio observacional**	**Y**	**Y**	**Y**	**N**	**Y**	**Y**	**Y**	**N**	**Y**	**Y**	**Y**	**Y**	**Y**	**Y**	**Y**	**N**	**Y**	**Y**	**N**	**N**	**Y**	**Y**	**N**	**Y**	**N**
Wang et al., 2010 [14]	**Estudio observacional**	**Y**	**Y**	**Y**	**N**	**Y**	**Y**	**Y**	**N**	**Y**	**N**	**Y**	**Y**	**Y**	**Y**	**N**	**N**	**Y**	**Y**	**N**	**N**	**Y**	**N**	**Y**	**Y**	**N**
Yaprak et al., 2020 [12]	**Estudio observacional**	**Y**	**Y**	**Y**	**N**	**Y**	**Y**	**Y**	**N**	**Y**	**Y**	**Y**	**Y**	**Y**	**Y**	**Y**	**N**	**Y**	**Y**	**N**	**N**	**Y**	**Y**	**N**	**Y**	**Y**
Han et al.,2014 [17]	**Estudio observacional**	**Y**	**Y**	**Y**	**N**	**Y**	**Y**	**Y**	**N**	**Y**	**Y**	**Y**	**Y**	**Y**	**Y**	**Y**	**N**	**Y**	**Y**	**N**	**N**	**B**	**Y**	**Y**	**Y**	**N**

Ikegami et al., 2008 [19]	**Estudio observacional**	**Y**	**Y**	**Y**	**N**	**Y**	**Y**	**Y**	**N**	**Y**	**N**	**Y**	**Y**	**Y**	**Y**	**N**	**N**	**Y**	**Y**	**N**	**N**	**Y**	**N**	**Y**	**Y**	**N**
Ji et al., 2017 [13]	**Estudio observacional**	**Y**	**Y**	**Y**	**N**	**Y**	**Y**	**Y**	**N**	**Y**	**Y**	**Y**	**Y**	**Y**	**Y**	**Y**	**N**	**Y**	**Y**	**N**	**N**	**Y**	**Y**	**N**	**Y**	**Y**
Yu et al., 2011 [16]	**Estudio de caso**																									
Lim et al., 2005 [18]	**Estudio observacional**																									
Ozsoy et al., 2011 [7]	**Estudio observacional**																									
